# Genetic association of type 2 diabetes and antidiabetic drug target with skin cancer

**DOI:** 10.3389/fmed.2024.1445853

**Published:** 2024-11-21

**Authors:** Juyan Zhao, Yu Zhang, Jianbo Li, Qi Li, Ziyue Teng

**Affiliations:** ^1^Department of Dermatology, Kunming City Maternal and Child Health Hospital, Kunming, China; ^2^Department of Dermatology, Ganmei Affiliated Hospital of Kunming Medical University, First People's Hospital of Kunming, Kunming, China; ^3^Department of Respiratory, Yan’an Hospital Affiliated to Kunming Medical University, Yan’an Hospital of Kunming City, Kunming, China

**Keywords:** antidiabetic drugs, Mendelian randomization, skin Cancer, type 2 diabetes, colocalization analysis

## Abstract

**Background:**

Several observational studies have suggested that type 2 diabetes (T2D) is a risk factor for skin cancer, and antidiabetic drugs may reduce skin cancer risk. Nevertheless, the findings remain ambiguous. This Mendelian randomization (MR) study aimed to investigate the causal association of T2D with skin cancer and evaluate the potential impact of antidiabetic drug targets on skin cancer.

**Methods:**

Genetic variants associated with glycated hemoglobin (HbA1c), Type 2 Diabetes (T2D), and antidiabetic drug targets (KCNJ11, ABCC8, PPARG, INSR, GLP1R, SLC5A2, and DPP4) were sourced from genome-wide association studies in the UK Biobank and the DIAMANTE consortium. Genetic summary statistics on skin cancer were obtained from the FinnGen consortium. MR analysis was primarily performed leveraging the inverse-variance weighted method, with additional sensitivity analyses conducted. Summary data-based MR (SMR) was utilized to further investigate the association between antidiabetic drug target gene expression and skin cancer. Colocalization analysis was carried out to verify the robustness of the results.

**Results:**

Genetically proxied elevated levels of HbA1c were found to be suggestively associated with a reduced risk of melanoma (OR: 0.886, 95% confidence interval (CI): 0.792–0.991, *p* = 0.0347). Additionally, genetically proxied T2D was notably associated with a lower risk of basal cell carcinoma (OR: 0.960, 95% CI: 0.928–0.992, *p* = 0.0147). The study also discovered that perturbation of the antidiabetic drug target SLC5A2 was significantly associated with an increased risk of basal cell carcinoma (for SLC5A2 perturbation equivalent to a 6.75 mmol/mol decrement in HbA1c: OR: 2.004, 95% CI: 1.270–3.161, *p* = 0.0027). However, this finding was not supported by colocalization analysis. Notably, no other drug target perturbations were found to be associated with skin cancer. Furthermore, SMR analysis failed to detect an association between antidiabetic drug target genes and skin cancer.

**Conclusion:**

The study suggests that higher HbA1c levels and T2D may be associated with a reduced risk of skin cancer. However, the results did not provide evidence to support the association between antidiabetic drug targets and skin cancer. Further evaluation of these drug targets is required to confirm the findings in this analysis.

## Introduction

Skin cancer is one of the most common cancers, mainly melanoma and non-melanoma skin cancer, with an increasing incidence rate, particularly in fair-skinned people. Non-melanoma skin cancer encompasses basal cell carcinomas (BCC) and squamous cell carcinomas (cSCC). Melanoma, the most lethal form of skin cancer, is estimated to occur in 25 new cases per 100,000 Europeans (1). BCC and cSCC, the two most prevalent forms of skin cancer, were expected to occur in 2.8 million and 1.5 million cases in the United States in 2019 (2). The majority of skin cancers are considered to be caused by the mutagenic impact of ultraviolet (UV) radiation. Given the intensification of UV radiation due to climate change, there is an urgent need for swift preventive measures against skin cancer (3). Identifying risk factors for skin cancer will aid in comprehending the development of the illness and guide efforts toward prevention and therapy.

Type 2 diabetes (T2D) is a chronic metabolic disease characterized by hyperglycemia, relative insulin deficiency, and insulin resistance. Its prevalence has increased steadily in recent decades, particularly among children and adolescents, posing a significant threat to global health (4). A crucial indicator for evaluating T2D glycemic management is glycated haemoglobin (HbA1c), which measures three-month mean blood glucose levels. Emerging research hints at a possible correlation between T2D and the risk of skin cancer. A cohort study showed a positive association between T2D and non-melanoma cancer incidence in the Finnish population (5). Another investigation in China observed a significant increase in the susceptibility to skin cancer exclusively among males with T2D (6). Nagore et al. (7) found that T2D was associated with greater melanoma aggressiveness at diagnosis. In addition, another cohort study also found significant rises in both cutaneous melanoma and non-melanoma skin malignancies merely among males (8). A meta-analysis indicates that T2D is a contributing factor for melanoma, while results from each study within the meta-analysis are inconclusive (9). Despite these findings, it is essential to acknowledge that epidemiological studies are subject to inherent limitations, such as selection bias, information bias, confounding variables, and the potential for reverse causality. Consequently, the definitive causal relationship between T2D and the risk of skin cancer remains uncertain and requires further research to elucidate.

Given the well-established association between T2D and multiple cancers, extensive research has been undertaken on the potential effect of antidiabetic medications on cancer risk. The classes of antidiabetic drugs used to treat T2D comprise biguanides (such as metformin), dipeptidyl peptidase 4 (DPP-4) inhibitors, sodium-glucose cotransporter 2 (SGLT-2) inhibitors, insulin/insulin analogues, glucagon-like peptide-1 (GLP-1) receptor agonists, sulfonylureas, and thiazolidinediones (10). Metformin-treated patients with T2D have been associated with a reduced risk of skin cancer in a cohort study (11). Two meta-analyses of observational studies and randomized controlled trials (RCTs), however, failed to reveal a statistically significant association between metformin and the risk of skin cancer in patients with T2D (12, 13). Recent cohort studies have indicated that DPP-4 inhibitors were associated with a reduced risk of melanoma but not non-melanoma skin cancer (14), while GLP-1 receptor agonists did not show any association with skin cancer (15) when compared with sulfonylureas. Moreover, Sung et al. (16) reported that SGLT-2 inhibitors were associated with a reduced cancer risk in comparison with DPP-4 inhibitors in a retrospective cohort study. Furthermore, a meta-analysis of RCTs uncovered that DPP-4 inhibitor treatment was associated with a reduced risk of skin cancer in patients with T2D (17). However, the use of SGLT-2 inhibitors, including Sotagliflozin, Empagliflozin, and Canagliflozin, was not found to be associated with the incidence of skin cancer in patients with T2D in a meta-analysis (18). Regarding thiazolidinediones, pioglitazone did not show conclusive evidence linking its use to the risk of skin cancer in individuals with T2D (19, 20). On the other hand, rosiglitazone, another thiazolidinedione, was suggested to potentially reduce the risk of non-melanoma skin cancer in Taiwanese patients with T2D (21). Hence, the impact of antidiabetic medications on skin cancer remains ambiguous.

Mendelian randomization (MR) is a novel approach that investigates the association between exposure and outcome by employing instrumental variables (IVs) as proxies for exposure (22, 23). MR analysis is referred to as a “natural randomized trial” since genetic variants (alleles) are assigned at random during meiosis and follow a temporal sequence. Thus, confounding and reverse causality biases can be minimized. Present MR approaches in drug target research are extensively employed to elucidate the causal relationship between antihypertensive, lipid-lowering, and antidiabetic drug targets and cancer and various chronic diseases. In the current study, we utilized MR analysis to explore the impact of T2D on skin cancer as well as the potential effects of antidiabetic drugs on skin cancer, aiming to provide evidence to guide clinical decisions in diabetes management and skin cancer prevention.

## Methods

**Figure 1:**
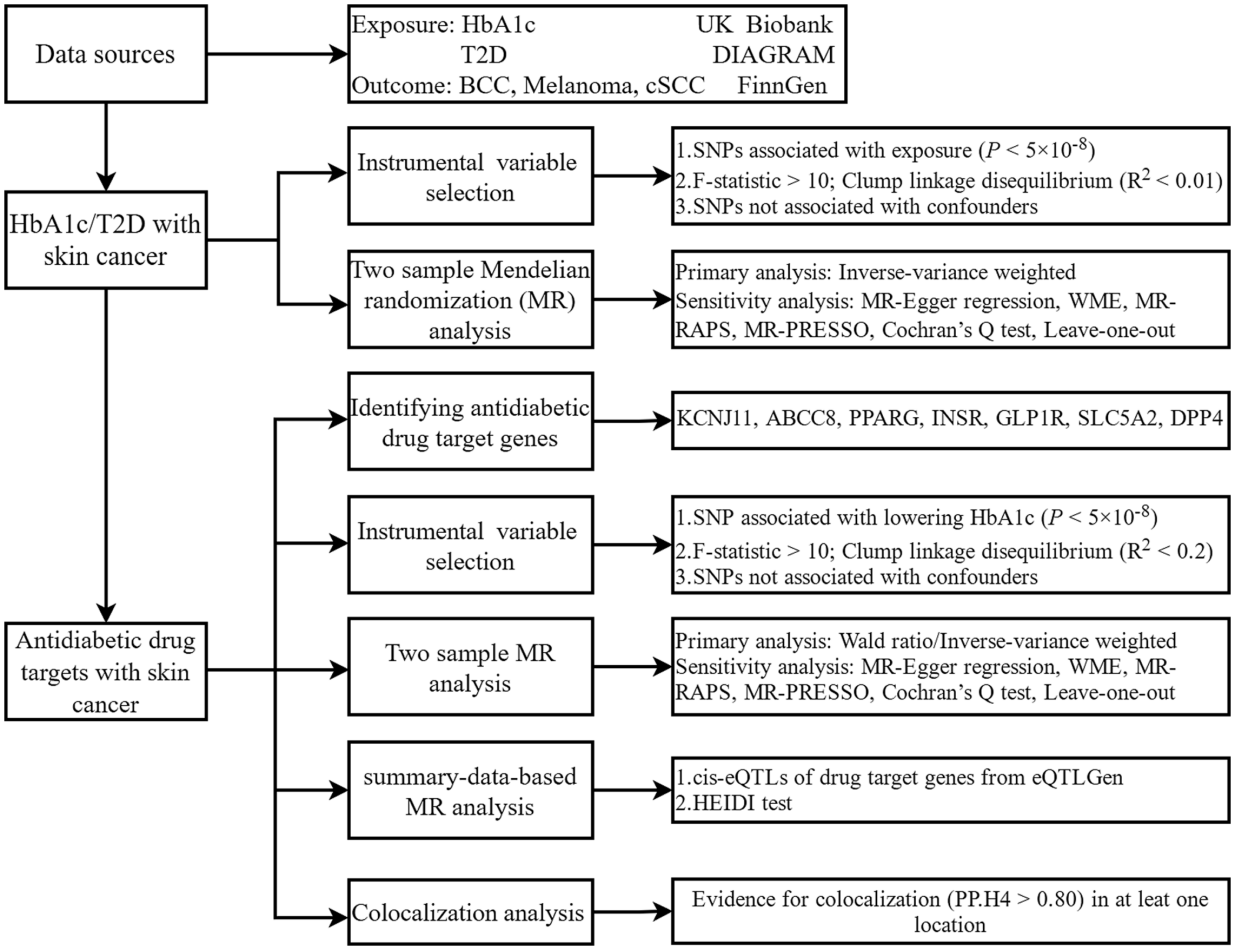
Study design. HbA1c, glycated haemoglobin; T2D, type 2 diabetes; BCC, basal cell carcinoma; cSCC, cutaneous squamous cell cancer; DIAGRAM, Diabetes Meta-Analysis of Trans-Ethnic association studies; SNP, single-nucleotide polymorphisms; WME, weighted median estimator; MR-RAPS, Mendelian randomization robust adjusted profile score; MR-RESSO, Mendelian randomization pleiotropy residual sum and outlier; HEIDI, heterogeneity in dependent instruments.

An overview of the research framework is depicted in Figure 1. The research is primarily structured into the subsequent stages: In the initial phase, the relationship between T2D, HbA1c, and skin cancer is investigated. The subsequent section explores the correlation between perturbation of the antidiabetic drug target and the risk of skin cancer. As a sensitivity analysis, the third section utilized the summary data-based MR (SMR) (24, 25) method, which assesses pleiotropic associations between gene expression and complex traits using summary-level data from expression quantitative trait loci (eQTL) studies, to clarify the association between genes targeted by antidiabetic drugs and skin cancer. In the end, colocalization analysis, which assesses whether two genetic signals occur at the same genomic location, verified the positive associations between antidiabetic drug targets and skin cancer.

### Data sources

Summary genetic association data for HbA1c, including 344,182 individuals composed of 13,586,180 single nucleotide polymorphisms (SNPs) were obtained from the UK Biobank consortium (Supplementary Table S1). The UK Biobank is a cohort study that follows a group of approximately 500,000 individuals who were between the ages of 40 and 69 as they were recruited in the years 2006 to 2010 (26). The genetic association dataset for T2D from the Diabetes Meta-Analysis of Trans-Ethnic Association Studies (DIAMANTE) consortium comprises 80,154 cases and 853,816 European controls (Supplementary Table S1) (27). Genetic summary data for cis-eQTL, defined as eQTL that regulate gene expression within the same genomic region, were obtained from eQTLGen, involving 31,684 subjects (28). As for the outcome, candidate genetic variants for skin cancer subtypes were acquired from the FinnGen consortium (R10), which is the latest published data (29). The dataset comprises three types of skin cancer: cutaneous squamous cell carcinoma (3,531 cases), basal cell carcinoma (20,506 cases), and melanoma (3,194 cases), along with 314,193 controls for each subtype (Supplementary Table S1) (29). There is no overlap between the exposure and outcome datasets.

### Genetic variant selection

To satisfy the three pivotal assumptions of MR, the IVs of HbA1c and T2D were meticulously validated as follows: (i) The SNP was strongly associated with exposure. SNP has a significance level with a *p-*value <5 × 10^−8^, and the independence of SNPs is guaranteed by the linkage disequilibrium (LD) clumping threshold (R^2^ < 0.01, windows distance = 10,000 kb), where LD denotes the non-random association of alleles across loci; (ii) The SNP was not associated with any known or unknown factors that could potentially influence the outcome. SNPs in relation to confounders (1, 30) or outcomes through LDtraits^1^ with *p* < 5 × 10^−8^ were ruled out (Supplementary Table S2) (31), and (iii) SNPs affect outcomes exclusively through exposure, yet proving this hypothesis poses a challenge. Moreover, to assess the strength of IVs, the F-statistic was calculated as *F* = (Beta^2^/SE^2^), where Beta is the effect size of the IV on the exposure, and SE denotes the corresponding standard error (32). An F-statistic <10 signifies weak IV bias, necessitating exclusion. Finally, the specific IVs chosen for HbA1c and T2D are detailed in Supplementary Tables S3–S8.

Seven categories of antidiabetic medications have been identified: biguanides (such as metformin), dipeptidyl peptidase 4 (DPP-4) inhibitors, sodium-glucose cotransporter 2 (SGLT-2) inhibitors, insulin/insulin analogues, glucagon-like peptide-1 (GLP-1) receptor agonists, sulfonylureas, and thiazolidinediones (10). The ChEMBL and DrugBank databases were utilized to identify the target genes of the active ingredients in these drugs (Supplementary Table S9). Due to target gene distinctions in the two databases and the obscurity surrounding its mechanism of action, metformin was excluded (33). To identify IVs for antidiabetic drug target perturbation, the criteria require SNPs within a ± 500 kb region of antidiabetic drug target genes must exhibit a significant association with both the reduction of HbA1c levels (*p* < 5 × 10^−8^) and the diminution of T2D risk. Then confirm the independence of SNPs (R^2^ < 0.2, clumping window = 500 kb). All the other criteria for IVs filtering are unaltered. Ultimately, the SNP information identified as IVs for antidiabetic drug target perturbation has been elaborated in Supplementary Tables S10–S12.

### Statistical analysis

The statistical power of MR studies was assessed utilizing an online tool^2^ (Supplementary Tables S13, S14) (34). In cases where outcome GWAS lacked exposure-related SNPs, proxy SNPs with high linkage disequilibrium (R^2^ > 0.80) with the SNPs were not leveraged. Strict data harmonization procedures were implemented to ensure that the effects of SNPs on outcomes and exposure aligned with the corresponding allele. For SNPs with different effect alleles due to strand differences, the strands were rectified. Reconciling palindromic SNPs could be challenging as they have the same allele on both strands, necessitating their exclusion. Later on, the Steiger filtering test was employed to ascertain the SNP’s directionality. SNPs with incorrect orientation were removed before conducting the MR analysis (Supplementary Tables S15–S17).

To assess the potential causal impact of HbA1c, T2D, and antidiabetic drug target perturbation on skin cancer, the Wald ratio (35) for one SNP or inverse-variance weighted (IVW, default: multiple random effects model) method (36) was the principal method adopted. Further, the MR-Egger regression (37), Weighted Median (38), MR-robust adjusted profile score (MR-RAPS) (39), and MR-pleiotropy residual sum and outlier (MR-PRESSO) (40) methods were implemented to enhance the reliability of the MR analysis findings. The MR-Egger intercept and Cochran’s Q tests were utilized to identify the existence of pleiotropy and heterogeneity, respectively. Leave-one-out analysis was carried out to determine whether a single SNP dominates the MR results.

We leveraged SMR approaches to investigate the associations between genes targeted by antidiabetic drugs and skin cancer. The top significant cis-eQTL loci with a *p*-value <5 × 10^−8^, located within a range of ±100 kb of seven antidiabetic drug-targeted genes, originated from the eQTGen consortium. Regrettably, no cis-eQTLs linked to the ABCC8 gene were identified. A *p*-value <0.0028 [0.05/ (6 genes*3 outcomes)] in SMR analysis indicates a statistically significant difference. The heterogeneity in dependent instruments (HEIDI) test was employed to distinguish pleiotropy from linkage. A *p*-value <0.01 demonstrates that associations found by SMR analysis could be explained by linkage. The SMR analysis employs the default parameter configuration. Additionally, a multi-SNP-based SMR analysis was carried out, adopting multiple SNPs with an LD value of R^2^ < 0.1 as IVs.

Bayesian colocalization analysis was implemented to determine the probability that two variables share a common causal genetic variation, as opposed to being caused by linkage disequilibrium. It rests on five presumptions, the specifics of which have already been laid out (41). Disregarding linkage disequilibrium and *p*-value filtering, we analyzed cis-eQTL loci located within a range of ±1 Mb from the SLC5A2 gene in HbA1c summary data, together with the corresponding basal cell carcinoma summary data. The execution utilized the default settings: p1: 1E-04, p2: 1E-04, and p12: prior probabilities of 1E-05. A significant colocalization was set at PP.H4 (posterior probability) > 0.80.

To account for multiple testing of HbA1c or T2D, a Bonferroni-corrected significance level of *p*-value <0.0167 (0.05/3) was utilized, while a *p*-value <0.0042 (0.05/4*3) was employed for perturbation in antidiabetic drug targets. A *p*-value falling between 0.05 and the Bonferroni-corrected *p*-value indicated a suggestive association. A two-sided *p*-value <0.05 was deemed statistically significant in other studies. All aforementioned statistical analyses were performed utilizing the “TwoSampleMR” (42) and “coloc” (43) packages in R (version 4.3.2) and SMR (24) software (version 1.3.1).

## Results

### HbA1c and T2D with skin cancer risk

**Figure 2:**
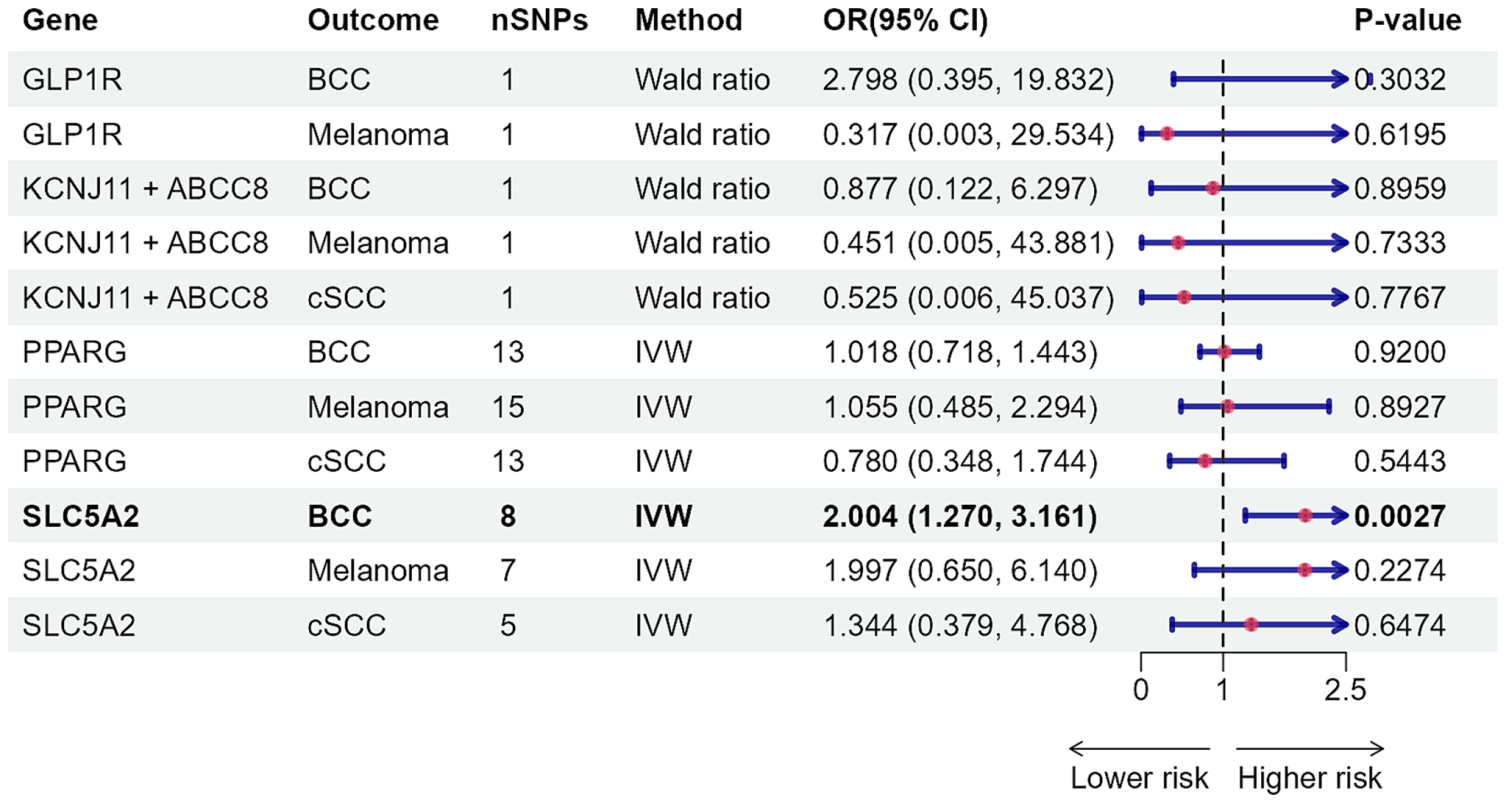
Mendelian randomization results of HbA1c with risk of skin cancer. nSNPs, number of single-nucleotide polymorphisms; OR, odds ratio; 95% CI, 95% confidence interval; HbA1c, glycated haemoglobin; BCC, basal cell carcinoma; cSCC, cutaneous squamous cell cancer; IVW, inverse-variance weighted; MR, Mendelian randomization; MR-RAPS, MR-robust adjusted profile score.

**Figure 3:**
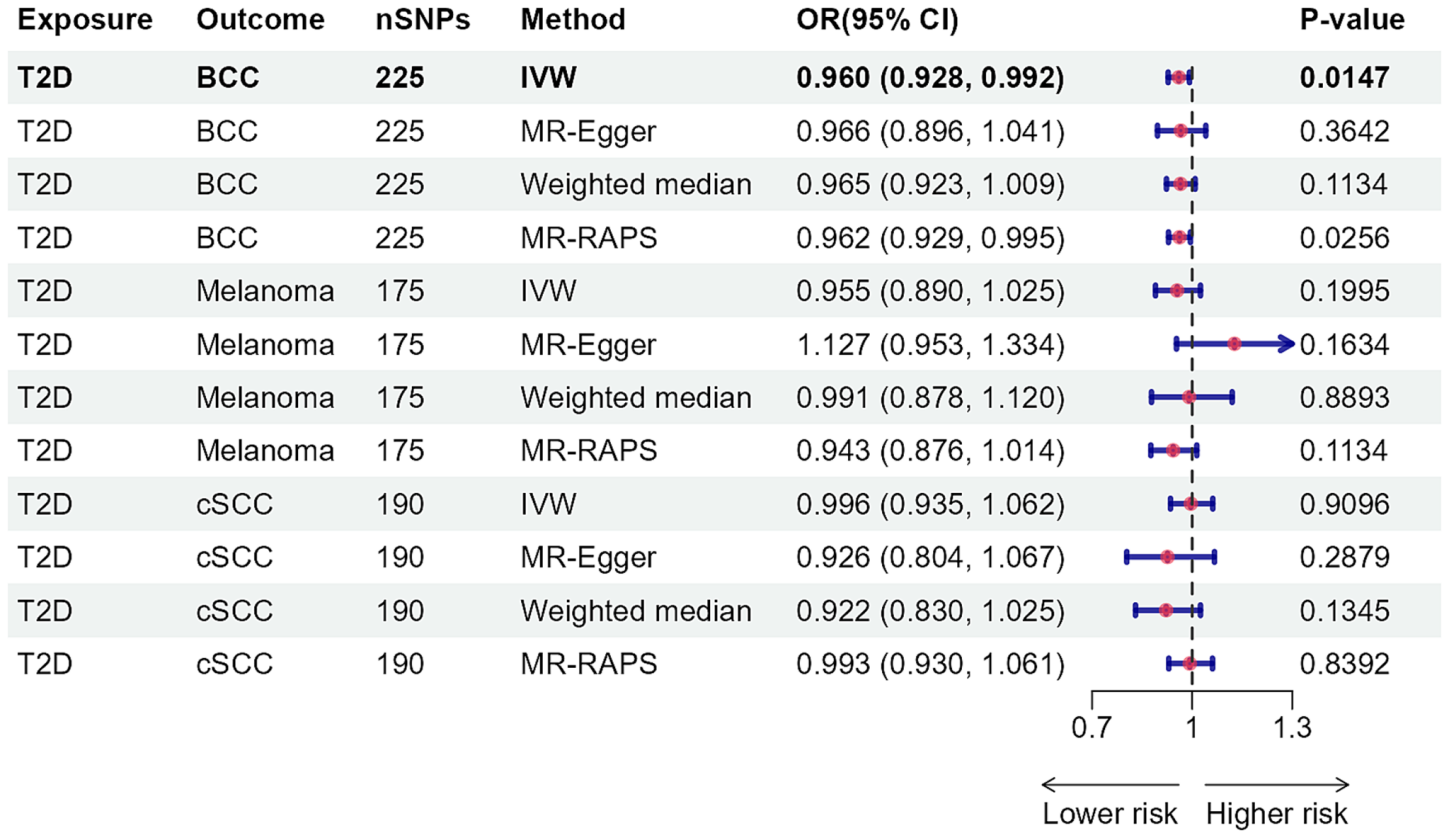
Mendelian randomization results for type 2 diabetes with risk of skin cancer. nSNPs, number of single-nucleotide polymorphisms; OR, odds ratio; 95% CI, 95% confidence interval; T2D, type 2 diabetes; BCC, basal cell carcinoma; cSCC, cutaneous squamous cell cancer; IVW, inverse-variance weighted; MR, Mendelian randomization; MR-RAPS, MR-robust adjusted profile score.

As Figures 2, 3 illustrated, genetically predicted increases in HbA1c levels, estimated based on genetic variants associated with HbA1c, were suggestively associated with a reduced risk of cutaneous melanoma (OR: 0.886, 95% confidence interval (CI): 0.792–0.991, *p* = 0.0347), while genetic liability to diabetes was nominally linked to a decreased risk of basal cell carcinoma (OR: 0.960, 95% CI: 0.928–0.992, *p* = 0.0147). No association was identified between genetically predicted HbA1c levels and other types of skin cancer (Supplementary Tables S18, S19). Further supplementary analyses assure the robustness of the current MR study results (Supplementary Tables S20–S22).

### Antidiabetic drug target perturbation with skin cancer risk

**Figure 4:**
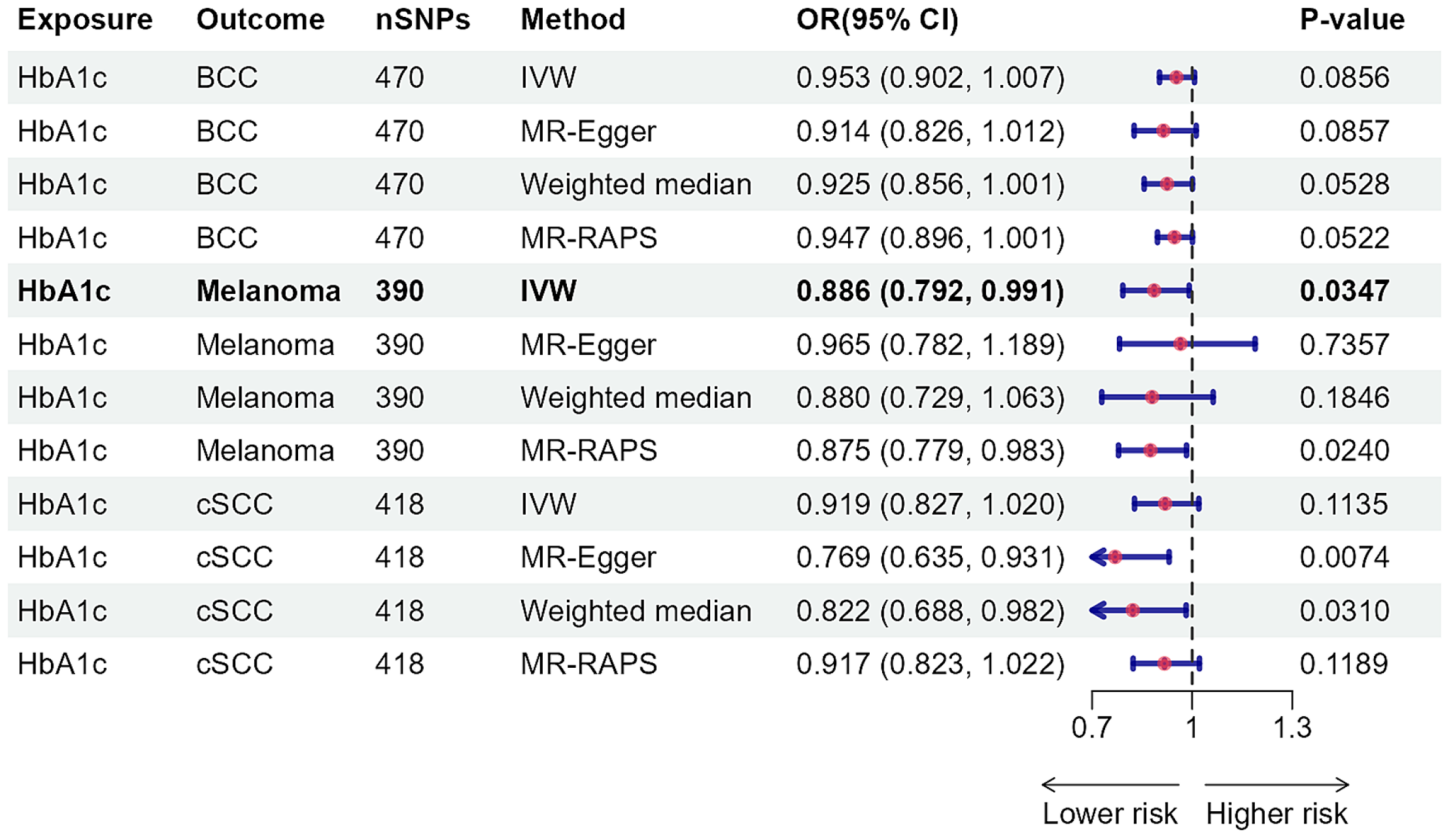
Association of genetically proxied perturbation of antidiabetic drug targets with risk of skin cancer. The OR and 95% CI indicate the effect estimates of an increase in skin cancer per SD unit (6.75 mmol/mol) reduction of HbA1c via antidiabetic drugs. nSNPs, number of single-nucleotide polymorphisms; OR, odds ratio; 95% CI, 95% confidence interval; BCC, basal cell carcinoma; cSCC, cutaneous squamous cell cancer; IVW, inverse-variance weighted.

Figure 4 depicts the causal effect of four classes of antidiabetic drugs on skin cancer risk. Genetically determined SLC5A2 perturbation, per standard deviation unit (SD) change of antidiabetic drug target perturbation equivalent to one SD unit (6.75 mmol/mol) of HbA1c lowering, was significantly associated with elevating the risk of BCC (OR: 2.004, 95% CI: 1.270–3.161, *p* = 0.0027) (Supplementary Table S23). The impacts of alternative MR approaches were consistent, albeit some failed to reach statistical significance (Supplementary Tables S23, S24). Neither Cochran’s Q test nor the MR-Egger intercept analysis yielded evidence of heterogeneity or pleiotropy (Supplementary Table S23). The leave-one-out approach confirmed the robustness of MR analysis findings (Supplementary Table S25). Insufficient evidence was found for the associations between genetically proxied GLP1R, KCNJ11, ABCC8, and PPARG perturbations and skin cancer risk.

### Antidiabetic drugs target gene expression with skin cancer risk

The SMR analysis results for antidiabetic drug target genes and skin cancer risk are shown in Supplementary Table S26. The analysis revealed no significant associations between the six targeted genes and the three skin cancer subtypes.

### Colocalization analysis

As indicated in Supplementary Table S27 and Supplementary Figure S1, the evidence supporting a shared causal variant between HbA1c levels and basal cell carcinoma at the SLC5A2 locus is relatively weak (PP.H4 < 0.80). Given that colocalization necessitates strong association signals from both traits to identify a shared causal variant, the lack of a robust association with basal cell carcinoma in this region suggests a limited capacity to detect colocalization.

## Discussion

This study systematically explores the causal effects of HbA1c, T2D, and antidiabetic drug targets on the risk of three distinct skin cancers. Two-sample MR analysis revealed a negative association between HbA1c levels and melanoma risk, as well as T2D and basal cell carcinoma risk. Moreover, the promoted impact of perturbation of the antidiabetic drug target SLC5A2 on the risk of basal cell carcinoma was also confirmed. However, our SMR analysis found no evidence linking the expression of antidiabetic drug target genes to skin cancer risk. Furthermore, there was limited evidence that HbA1c and basal cell carcinoma share common causal variants in SLC5A2. Besides, our findings did not support the antidiabetic drug target perturbations, apart from SLC5A2, in relation to skin cancer risk.

Research exploring the link between HbA1c levels and melanoma is currently limited. An MR analysis has demonstrated that participants with higher levels of fasting plasma glucose, glycated haemoglobin, 2-h plasma glucose, and T2D had a decreased risk of melanoma, which aligns with our finding (44). Another MR study on T2D and melanoma yielded identical findings (45). A nationwide investigation in Sweden unveiled that individuals with familial T2D, totaling 125,126 patients, were notably associated with a reduced risk of melanoma compared to the general population (46). Similarly, a nationwide study in Australia revealed that 872,706 individuals with T2D had a reduced melanoma risk (47). Nonetheless, a comprehensive cohort study of 4,501,578 U.S. veterans confirmed that those with diabetes, particularly men, were at a higher risk of melanoma (48). With regard to the association of T2D with BCC, the current research is constrained. A Finnish cohort study revealed a significant increase in non-melanoma cancer incidence among individuals with diabetes (5). Another retrospective cohort study implied that Lithuanian men with T2D had a higher risk of both melanoma and non-melanoma skin cancer (8). Potential causes for the contradictory results might be ascribed to racial disparities, the limited size of the sample, and the bias of confounders and reverse causality. Research into how T2D may reduce skin cancer risk remains limited. In T2D, prolonged hyperglycemia and insulin resistance often result in elevated levels of insulin and insulin-like growth factors (IGF), which activate the insulin/IGF signaling pathway. Ultraviolet (UV) radiation exposure, a key factor in non-melanoma skin cancer development, causes DNA damage in skin cells. Interestingly, low IGF-1 receptor activity within these cells might weaken their ability to respond to UV-induced damage (49), potentially facilitating the progression of skin cancer. This interplay between T2D, UV radiation, and the IGF signaling pathway may partly explain the observed association.

In reconciling the discrepancies observed between MR and traditional observational studies regarding the association of T2D and antidiabetic drug targets with skin cancer risk, several factors merit consideration. First, MR studies leverage genetic instruments that mitigate the influences of confounding and reverse causation, the latter of which can significantly bias findings in case–control studies, where the outcome may inadvertently affect the exposure assessment. Additionally, traditional observational studies often contend with limited sample sizes, which can restrict statistical power and increase susceptibility to random error. These studies may also be confounded by unmeasured variables, such as lifestyle factors or environmental exposures, that may differentially impact both T2D and skin cancer risk. Furthermore, the MR approach reflects lifelong genetic predisposition to T2D, contrasting with observational studies that typically capture short-term clinical diagnoses, wherein the duration of diabetes and treatment history may vary considerably. Such differences in exposure characterization are pivotal in elucidating the inconsistencies in risk estimates.

While our MR study unveiled a noteworthy interplay between genetically proxied SLC5A2 perturbation and an elevated risk of basal cell cancer, the colocalization analysis failed to substantiate this finding. Notwithstanding contradicting one prior study (16), our findings align with a meta-analysis that did not support the effect of SGLT-2 inhibitors targeting SLC5A2 on the risk of skin cancer in patients with T2D (18). Besides, our investigation did not reveal any association between perturbations in other targets of antidiabetic drugs and an increased risk of skin cancer, which parallels previous meta-analyses. Two meta-analyses, encompassing both observational studies and RCTs, reinforced the lack of a statistically significant association between the use of metformin and the risk of skin cancer in persons with T2D (12, 13). Furthermore, no conclusive evidence supports an association between pioglitazone, a thiazolidinedione, and skin cancer risk in T2D patients (19, 20). Currently, there is limited investigation into the mechanisms by which SGLT-2 inhibition via the SLC5A2 target may influence skin cancer risk. We propose two hypotheses: SGLT-2 inhibitors may induce fixed drug eruptions (50) that compromise the skin barrier and alter pigmentation, potentially elevating the risk of skin cancer. Additionally, SGLT-2 inhibitors are capable of promoting skin wound repair (51) through cellular proliferation and angiogenesis, processes crucial for healing that may support cancer cell growth.

This study possesses several notable strengths. We conducted a comprehensive investigation utilizing two-sample MR and SMR analyses to explore the potential association between antidiabetic drug targets and the risk of skin cancer. The adoption of the MR design enables the effective mitigation of confounding and reverse causality biases. Moreover, it is worth noting that the study participants were exclusively of European ancestry, a deliberate selection aimed at minimizing the bias arising from population stratification. Ultimately, the F-statistic value ascertained that the presence of weak IVs bias is highly unlikely.

Some limitations need to be acknowledged in this study. First, MR estimates reflect lifetime drug exposure, while drugs often take action for a short timeframe. Consequently, the effect sizes in our investigation may not be directly comparable to those reported in experimental or observational studies (52). Second, this study focused solely on predicting the target effects of antidiabetic drugs by considering drug targets with specific pharmacological mechanisms, while failing to identify off-target impacts. Third, the MR results on SLC5A2 perturbation do not match up with the colocalization analysis. This discrepancy may be attributed to the divergent methodologies employed by the two approaches. While MR searches for exposure-related variants, colocalization adopts a more conservative approach, requiring the association of causal variants with both exposure and outcome (53). The colocalization analysis revealed a tenuous genetic association between the genetic variant and basal cell carcinomas, which may explain the observed diminished colocalization. Fourth, owing to the lack of reliable genetic tools, we were unable to evaluate the effects of certain antidiabetic drug target perturbations (such as INSR and DPP4) on skin cancer. Fifth, we acknowledge that our study may have been limited by inadequate statistical power, yielding a null effect estimate. Furthermore, our analysis did not account for gene–environment or gene–gene interactions, as well as the linear and time-dependent effects of antidiabetic drug targets on skin cancer risk. At last, the sample for this study was exclusively drawn from the European population, thereby limiting the generalizability of our findings. It is important to note that genetic variants associated with T2D may exhibit significant frequency variations among non-European populations. Additionally, environmental factors such as ultraviolet exposure and skin type may exert differential effects on skin cancer risk across various ethnic groups. Consequently, further research involving diverse populations is imperative to achieve a comprehensive understanding of the complex interplay between T2D and skin cancer.

In conclusion, our MR studies have demonstrated that genetically proxied elevations in HbA1c levels are suggestively associated with a reduced risk of melanoma, and genetically proxied T2D is significantly associated with a decreased risk of basal cell carcinoma. Additionally, we identified that the perturbation of the antidiabetic drug target SLC5A2 has a significant impact on elevating the risk of basal cell carcinoma, yet this was not supported by colocalization evidence. Furthermore, we found limited evidence of an association between the target perturbation of other antidiabetic drugs and the risk of skin cancer. Finally, SMR analysis also failed to uncover any association between antidiabetic drug target gene expression and skin cancer risk.

## Data Availability

The original contributions presented in the study are included in the article/Supplementary material, further inquiries can be directed to the corresponding author.

## Footnotes

^1^ https://ldlink.nci.nih.gov/?tab=ldtrait

^2^ https://shiny.cnsgenomics.com/mRnd/

## Supplementary material

SUPPLEMENTARY FIGURE S1 Regional Manhattan plot of associations of SLC5A2 with the risk of basal cell carcinoma. Rs57611503 (purple diamond) represents the sentinel SNP. SNPs within ±1 Mb of the antidiabetic drug target SLC5A2 quantitative trait locus were included. BCC, basal cell carcinoma.
