# Supplementary figures and images for: Genetic association of type 2 diabetes and antidiabetic drug target with skin cancer
Source: Front Med (Lausanne). 2024 Nov 21;11:1445853. doi: 10.3389/fmed.2024.1445853 (PMC11617162; doi:10.3389/fmed.2024.1445853)

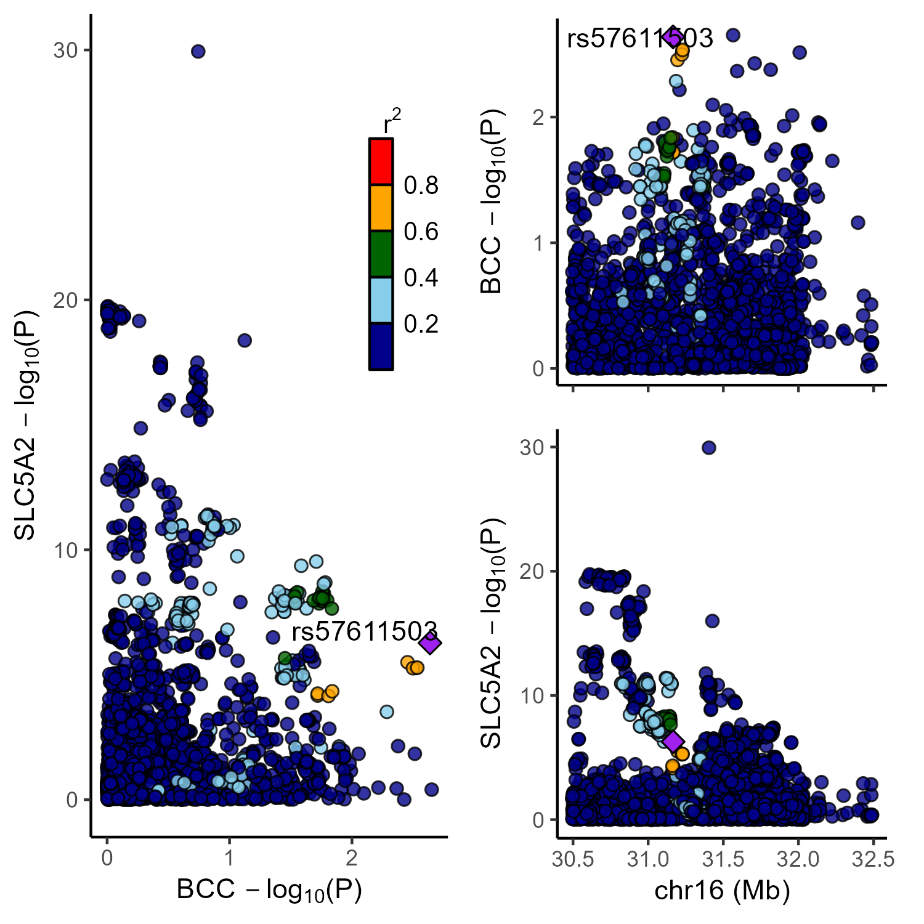

Supplement: SUPPLEMENTARY FIGURE S1 — Regional Manhattan plot of associations of SLC5A2 with the risk of basal cell carcinoma. Rs57611503 (purple diamond) represents the sentinel SNP. SNPs within ±1 Mb of the antidiabetic drug target SLC5A2 quantitative trait locus were included. BCC, basal cell carcinoma. [file Image_1.TIF]
